# Modulatory Effects of Fractalkine on Inflammatory Response and Iron Metabolism of Lipopolysaccharide and Lipoteichoic Acid-Activated THP-1 Macrophages

**DOI:** 10.3390/ijms23052629

**Published:** 2022-02-27

**Authors:** Edina Pandur, Kitti Tamási, Ramóna Pap, Gergely Jánosa, Katalin Sipos

**Affiliations:** Department of Pharmaceutical Biology, Faculty of Pharmacy, University of Pécs, Rókus Str. 2, H-7624 Pécs, Hungary; kitti.tamasi@aok.pte.hu (K.T.); pap.ramona@pte.hu (R.P.); janosa.gergely@gytk.pte.hu (G.J.); katalin.sipos@aok.pte.hu (K.S.)

**Keywords:** macrophage, fractalkine, inflammation, iron metabolism, cell signalling, cytokine, lipopolysaccharide, lipoteichoic acid

## Abstract

Fractalkine (CX3CL1) acts as a chemokine as well as a regulator of iron metabolism. Fractalkine binds CX3CR1, the fractalkine receptor on the surface of monocytes/macrophages regulating different intracellular signalling pathways such as mitogen-activated protein kinase (MAPK), phospholipase C (PLC) and NFκB contributing to the production of pro-inflammatory cytokine synthesis, and the regulation of cell growth, differentiation, proliferation and metabolism. In this study, we focused on the modulatory effects of fractalkine on the immune response and on the iron metabolism of *Escherichia coli* and *Pseudomonas aeruginosa* lipopolysaccharides (LPS) and *Staphylococcus aureus* lipoteichoic acid (LTA) activated THP-1 cells to get a deeper insight into the role of soluble fractalkine in the regulation of the innate immune system. Pro-inflammatory cytokine secretions of the fractalkine-treated, LPS/LTA-treated, and co-treated THP-1 cells were determined using ELISArray and ELISA measurements. We analysed the protein expression levels of signalling molecules regulated by CX3CR1 as well as hepcidin, the major iron regulatory hormone, the iron transporters, the iron storage proteins and mitochondrial iron utilization. The results showed that fractalkine treatment alone did not affect the pro-inflammatory cytokine secretion, but it was proposed to act as a regulator of the iron metabolism of THP-1 cells. In the case of two different LPS and one type of LTA with fractalkine co-treatments, fractalkine was able to alter the levels of signalling proteins (NFκB, PSTAT3, Nrf2/Keap-1) regulating the expression of pro-inflammatory cytokines as well as hepcidin, and the iron storage and utilization of the THP-1 cells.

## 1. Introduction

Monocytes, the innate immune cells of the human body, can be activated by various extracellular stimuli (e.g., bacterial components, foreign particles, inflammatory cytokines and other mediators, etc.) [1]. The monocytes can differentiate into two major types of macrophages, M1 and M2 [2]. Monocytes/macrophages can be differentiated to the M1 type by Gram-negative endotoxin lipopolysaccharide (LPS) or Gram-positive cell wall component lipoteichoic acid (LTA). M1 macrophages produce pro-inflammatory cytokines (e.g., IL-1β, IL-6 or TNFα) and mediate antimicrobial defence and tissue destruction [2]. Macrophages also work as antigen-presenting cells providing immunostimulation for helper T cells [3]. M2 macrophages can be developed by anti-inflammatory cytokines and glucocorticoids mediating anti-inflammatory signals, tissue repair and angiogenesis [4].

Gram-negative and Gram-positive bacteria activate monocytes via an interaction between bacterial cell wall components LPS or LTA and specific cell surface receptors, such as toll-like receptors (TLR) [5,6]. Activation of TLR4 by LPS and TLR2 by LTA triggers MAPK (mitogen-activated protein kinase) and NFκB signalling pathways. The latter pathway is crucial in the transactivation of the pro-inflammatory cytokine encoding genes [7].

According to the literature, fractalkine (CX3CL1) acts as a chemokine as well as an inflammatory mediator and a regulator of the iron metabolism of various cells [8,9,10]. Fractalkine is expressed by many types of cells (e.g., neurons, astrocytes, endometrial cells and immune cells) and is also present on the surface of the endothelial cells of the blood vessels as a membrane-bound form, which provides an anchoring site for the macrophages and lymphocytes [11,12,13,14,15,16]. Fractalkine also can be secreted as a soluble form by the proteolytic cleavage of the membrane-bound fractalkine acting as an inflammatory mediator [17,18]. Both forms of fractalkine are able to bind and modify the activity of fractalkine receptor (CX3CR1) expressed by various cell types including microglia, neurons, endometrial cells, trophoblasts, lymphocytes and monocytes/macrophages [8,15,19,20,21]. CX3CR1 regulates three downstream signalling pathways: MAPK, PLC (phospholipase C) and NFκB contributing to the pro-inflammatory cytokine synthesis as well as cell growth, differentiation, proliferation and metabolism [22,23].

One strategy of the immune system for inhibiting bacterial cell proliferation is the sequestration of iron from the circulation by macrophages, which inhibits bacterial cell proliferation [24,25]. Changes in the iron metabolism of macrophages by fractalkine may increase the iron content of the cells, which can contribute to the elimination of bacteria by iron-mediated reactive oxygen species [26,27,28].

In the present work, we focused on the modulatory effect of soluble fractalkine on the production of the pro-inflammatory cytokines and on the iron metabolism of *Escherichia coli* and *Pseudomonas aeruginosa* LPS and *Staphylococcus aureus* LTA-activated THP-1 cells for a better understanding of the role of soluble fractalkine in the regulation of the innate immune system. We found that fractalkine alone did not affect the pro-inflammatory cytokine secretion but was proven to act as a regulator of the iron metabolism of THP-1 cells. In the case of LPS or LTA and fractalkine co-treatments, fractalkine was able to modify the activity of the intracellular signalling pathways (NFκB, PSTAT3, Nrf2/Keap-1) regulating the expression of pro-inflammatory cytokines as well as the iron regulatory hormone hepcidin, and the iron storage and utilization of the THP-1 cells.

## 2. Results

### 2.1. Effects of Fractalkine on the Pro-Inflammatory Cytokine Expression of LPS or LTA Activated THP-1 Cells

**Figure 1:**
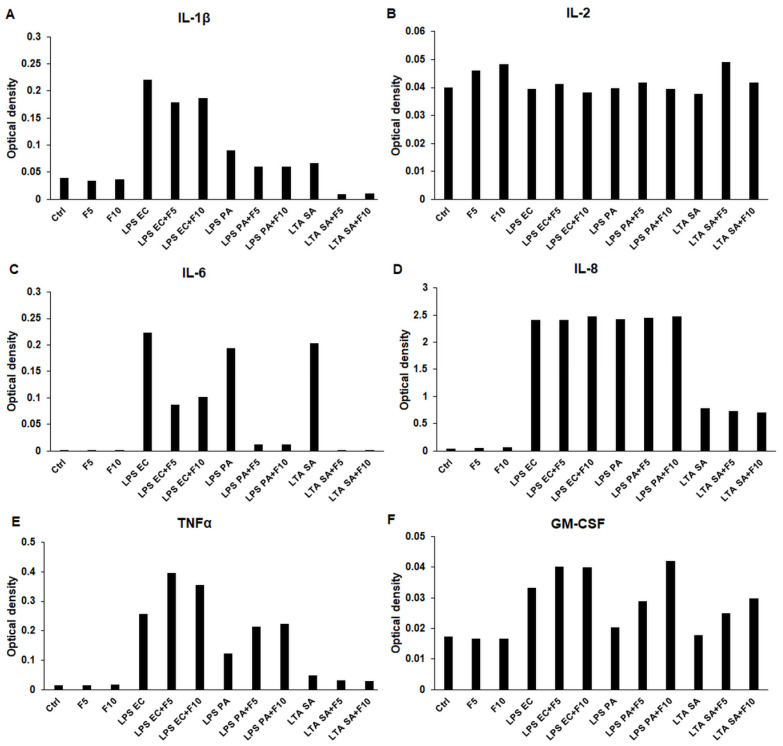
ELISArray determination of pro-inflammatory cytokine production of *E. coli* or *P. aeruginosa* LPS and *S. aureus* LTA and/or fractalkine-treated THP-1 cells. The arrays were carried out using pooled cell culture supernatants obtained from the same treatment types of four independent experiments (*n* = 4). In the case of co-treatments, both the LPS or LTA and fractalkine were added at the same time to the cells. Supernatants were collected after 24 h of treatments. The columns show the optical density of the average Il-1β (**A**), IL-2 (**B**), IL-6 (**C**), IL-8 (**D**), TNFα (**E**) and GM-CSF (**F**) production of THP-1 cells. Abbreviations: F5—fractalkine 5 ng/mL; F10—fractalkine 10 ng/mL; LPS EC—*E. coli* lipopolysaccharide; LPS PA—*P. aeruginosa* lipopolysaccharide; LTA SA—*S. aureus* lipoteichoic acid.

To reveal which pro-inflammatory cytokines are produced and altered by the activation of THP-1 cells and by fractalkine treatments, ELISArray was carried out, which is suitable for the parallel determination of six types of cytokines (IL-1β, IL-2, IL-6, IL-8, TNFα and GM-CSF) from cell culture supernatants (Figure 1). The arrays were carried out using pooled cell culture supernatants of four experiments. The results showed that fractalkine alone did not alter the cytokine production (except in the case of IL-2, which increased after fractalkine treatment) of THP-1 cells, suggesting that fractalkine without the presence of an inflammatory mediator did not affect the inflammatory response of THP-1 cells (Figure 1). However, fractalkine was able to increase the production of TNFα of LPS co-treated cells meanwhile decreasing it in the case of LTA/fractalkine co-treatment (Figure 1E). Fractalkine also increased GM-CSF secretion of the LPS as well as the LTA-activated THP-1 cells in a concentration-dependent manner (Figure 1F). In spite of this, fractalkine decreased both IL-1β and IL-6 production of co-treated cells compared to LPS or LTA treatments alone (Figure 1A,C). IL-2 showed less dramatic changes and IL-8 production was not altered in the case of co-treatments with LPS or LTA and fractalkine (Figure 1B,D).

**Figure 2:**
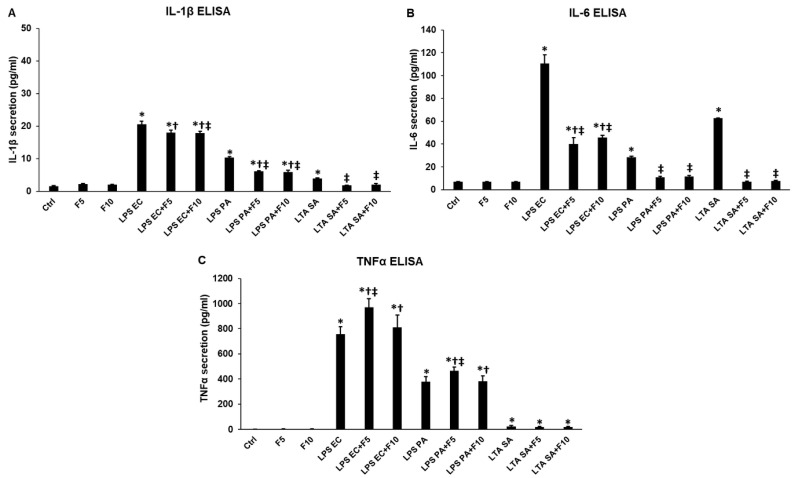
ELISA measurement of IL-1β (**A**), IL-6 (**B**) and TNFα (**C**) pro-inflammatory cytokine secretion of *E. coli* or *P. aeruginosa* LPS and *S. aureus* LTA and/or fractalkine-treated THP-1 cells. Pro-inflammatory cytokine secretions were determined using human IL-1β, IL-6 and TNFα specific ELISA kits according to the manufacturer’s instructions. The bars represent mean values and error bars represent standard deviation (SD) for three independent determinations (*n* = 3). ELISA measurements were made in triplicate in each independent experiment. Statistical significance was determined by two-way ANOVA (considering the number of the categorical variables) followed by Scheffe’s post hoc test. Asterisks indicate *p* < 0.05 compared to the control. Cross marks *p* < 0.05 compared to the fractalkine treatment. Double cross shows *p* < 0.05 compared to the LPS or LTA treatments, respectively. Abbreviations: F5—fractalkine 5 ng/mL; F10—fractalkine 10 ng/mL; LPS EC—*E. coli* lipopolysaccharide; LPS PA—*P. aeruginosa* lipopolysaccharide; LTA SA—*S. aureus* lipoteichoic acid.

We also performed ELISA measurements for the determination of the concentration changes of secreted IL-1β, IL-6 and TNFα cytokines. In parallel with the ELISArray measurements, fractalkine treatments of THP-1 cells did not affect the secretion of the examined pro-inflammatory cytokines (Figure 2). In the case of co-treatments, both fractalkine concentrations significantly decreased IL-1β (LPS EC/FKN 17.89 ± 0.51 pg/mL, *p* = 0.0001; LPS PA/FKN 5.89 ± 0.54 pg/mL, *p* = 0.00014; LTA SA/FKN 1.86 ± 0.01 pg/mL, *p* = 0.00011) and IL-6 (LPS EC/FKN 45.57 ± 1.89 pg/mL, *p* = 0.00012; LPS PA/FKN 10.77 ± 0.87 pg/mL, *p* = 0.0001; LTA SA/FKN 6.95 ± 0.44 pg/mL, *p* = 0.00001) production compared to LPS (IL-1β EC 20.61 ± 0.86 pg/mL; PA 10.34 ± 0.36 pg/mL; IL-6 EC 110.59 ± 7.45 pg/mL; PA 28.54 ± 0.89 pg/mL) and LTA (IL-1β 3.92 ± 0.21 pg/mL; IL-6 62.52 ± 0.41 pg/mL) treatments (Figure 2A,B). Meanwhile, TNFα secretion was elevated due to fractalkine/LPS co-treatments (970 ± 69.58 pg/mL, *p* = 0.0002; 465.7 ± 30.14 pg/mL, *p* = 0.00018) compared to LPS EC (755.9 ± 58.65 pg/mL) and LPS PA (377.9 ± 40.26 pg/mL) treatments (Figure 2C). On the other hand, fractalkine did not change TNFα secretion when LTA was used in the co-treatment (Figure 2C). These results suppose that fractalkine may act differently on the expression of the examined pro-inflammatory cytokines possibly via the regulation of distinct intracellular signalling pathways depending on the inflammatory mediator.

### 2.2. Fractalkine Affects Differently the Expression of CX3CR1 Receptor and the Downstream Signalling Pathways in LPS or LTA Treated THP-1 Cells

Soluble fractalkine binds to its receptor, CX3CR1, and directly or indirectly regulates different intracellular signalling pathways, e.g., NFκB, MAPK, STAT3, Nrf2, with which it is able to modify both the inflammatory response as well as the iron metabolism of the cells [22,23].

**Figure 3:**
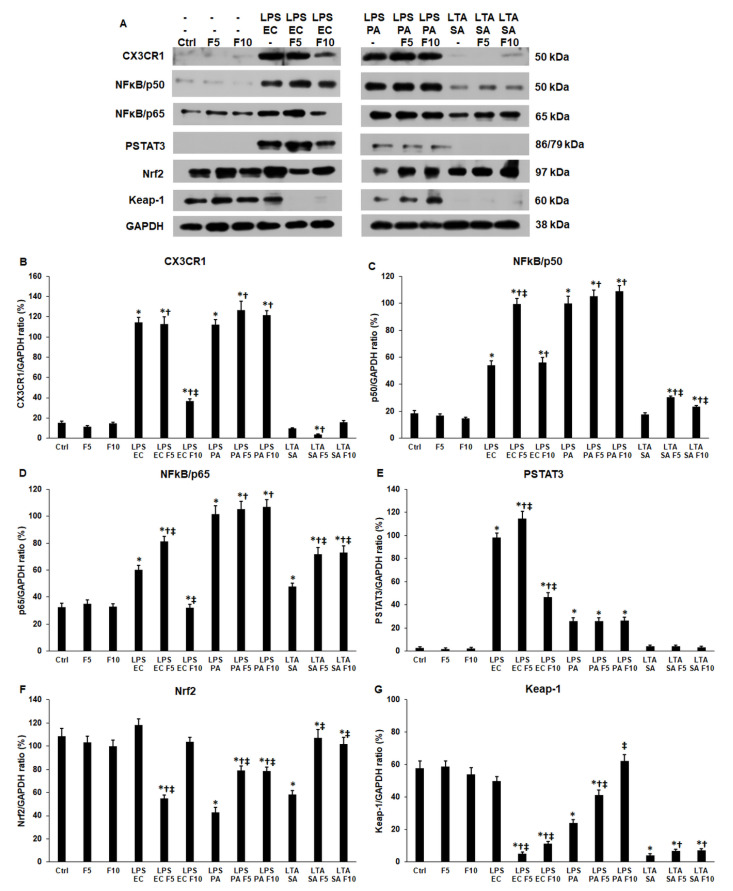
Western blot analyses of CX3CR1 (fractalkine receptor) and the downstream signalling proteins (NFκB/p50, NFκB/p65, phospho-STAT3, Nrf2 and Keap-1) regulating cytokine expression and iron metabolism of *E. coli* or *P. aeruginosa* LPS and *S. aureus* LTA and/or fractalkine-treated THP-1 cells. The blots were probed with anti-CX3CR1, anti-p50, anti-p65, anti-phospho-STAT3, anti-Nrf2 and anti-Keap-1 polyclonal rabbit antibodies according to the manufacturer’s protocols. GAPDH was used as loading control on each membrane. (**A**) Western blot analyses of the examined proteins in THP-1 cells. (**B**–**G**) Optical density analyses of CX3CR1, p50, p65, PSTAT3, Nrf2 and Keap-1 proteins. The columns represent mean values and error bars represent standard deviation (SD) of three independent experiments (*n* = 3). Statistical significance was determined by two-way ANOVA (considering the number of the categorical variables) followed by Scheffe’s post hoc test. Asterisks indicate *p* < 0.05 compared to the control. Cross marks *p* < 0.05 compared to the fractalkine treatment. Double cross shows *p* < 0.05 compared to the LPS or LTA treatments, respectively. Abbreviations: F5—fractalkine 5 ng/mL; F10—fractalkine 10 ng/mL; LPS EC—*E. coli* lipopolysaccharide; LPS PA—*P. aeruginosa* lipopolysaccharide; LTA SA—*S. aureus* lipoteichoic acid.

We found that the utilised concentrations (5 and 10 ng/mL) of soluble fractalkine on THP-1 cells did not alter the expression level of the examined proteins (Figure 3A), suggesting that fractalkine without an additional inflammatory signal did not change the activation of the downstream signalling pathways. In spite of this, fractalkine was able to modify the expression level of the signalling proteins during inflammation. When 10 ng/mL fractalkine was added together with LPS EC, the CX3CR1 level (36.8 ± 2.5%, *p* = 0.0001) decreased significantly compared to LPS EC treatment (114.66 ± 4.58%), but in the case of LPS PA treatment, fractalkine was ineffective on CX3CR1 protein expression (Figure 3A,B). Moreover, THP-1 cells treated with LTA SA showed a decreased CX3CR1 level (3.37 ± 0.87%, *p* = 0.0002) by adding 5 ng/mL fractalkine (Figure 3A,B). These results suggest that fractalkine acts differently in the case of Gram-positive and Gram-negative bacterial infections and the bacterial origin of LPS operates as a determining factor (*E. coli* or *P. aeruginosa*).

The p50 and p65 protein levels followed the alterations of the CX3CR1 protein in the case of fractalkine and LPS EC (99.58 ± 4.15%, *p* = 0.0001) and LPS PA (105.26 ± 4.75%, *p* = 0.0002) co-treatments, but in the case of LTA SA treatment, fractalkine increased p50 (30.33 ± 1.11%, *p* = 0.0018) and p65 (71.79 ± 4.75%, *p* = 0.0001) protein levels compared to LPS (p50 EC 54 ± 3.25%, p50 PA 100 ± 5.21%; p65 EC 60 ± 3.54%, p65 PA 101.72 ± 5.21%) or LTA (p50 17.68 ± 1.23%, p65 47.62 ± 2.64%) treatments alone (Figure 3A,C,D).

LPS EC and 5 ng/mL fractalkine co-treatment increased (114.52 ± 6.54%, *p* = 0.00011), while LPS EC with 10 ng/mL fractalkine decreased (46.4 ± 4.25%, *p* = 0.00013) the PSTAT3 protein level (Figure 3A,E), which were consistent with p50 and p65 protein levels. In the case of the other two bacterial cell wall components, fractalkine did not affect PSTAT3 expression (Figure 3A,E).

The Nrf2 transcription factor level increased in the case of LPS EC and 5 ng/mL fractalkine co-treatments (54.77 ± 3.22%, *p* = 0.0002) but did not change using 10 ng/mL fractalkine (103.52 ± 3.87%, *p* = 0.057) compared to LPS EC (118.33 ± 4.98%) (Figure 3A,F). Interestingly, in the case of LPS PA or LTA SA and fractalkine co-treatments (PA 78.94 ± 4%, *p* = 0.0001; SA 107.26 ± 7%, *p* = 0.00011), Nrf2 protein levels were significantly elevated compared to LPS PA (43.1 ± 4.11%) or LTA SA (58.5 ± 3.25%) (Figure 3A,F). We also examined the inhibitor of the Nrf2 transcription factor, Keap-1. The Keap-1 protein level decreased in the case of LPS EC and fractalkine co-treatments (5.14 ± 1%, *p* = 0.0001) but increased in the case of LPS PA and fractalkine co-treatments (62.5 ± 3.54%, *p* = 0.0002) compared to the LPS EC (50 ± 2.54%) and PA (24.13 ± 2%) treatments (Figure 3A,G). Fractalkine was less effective on the LTA SA-treated THP-1 cells; significant change was not found in the Keap-1 protein level (Figure 3A,G).

Based on these findings, it seems that fractalkine has distinct effects on the signalling proteins depending on the inflammatory mediator used for the activation of THP-1 cells, and these effects can be dose dependent.

### 2.3. Fractalkine Increases HAMP mRNA Expression but Not Hepcidin Secretion of THP-1 Cells

**Figure 4:**
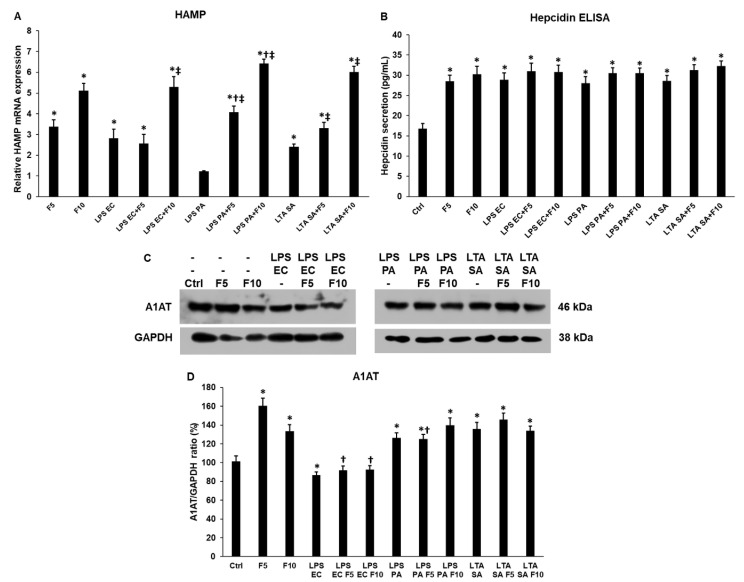
Real time PCR and ELISA measurements of hepcidin expression and Western blot analysis of alpha-1 antitrypsin (A1AT) of *E. coli* or *P. aeruginosa* LPS and *S. aureus* LTA and/or fractalkine-treated THP-1 cells. Real-time PCR was carried out with SYBR Green protocol and β-actin housekeeping gene was used as internal control for the normalization of the reaction. The relative expression of untreated control cells was regarded as 1. Secreted hepcidin concentration of the culture media was determined with a human hepcidin ELISA kit according to the instructions of the manufacturer. For the WB, the cells were collected and lysed. The same amount of protein from each lysate was used for the analysis. (**A**) mRNA expression levels of HAMP of THP-1 cells treated with *E. coli* or *P. aeruginosa* LPS and *S. aureus* LTA and/or fractalkine (**B**) Secreted hepcidin concentration of THP-1 cells. (**C**) Western blot analysis of A1AT protein of THP-1 cells. (**D**) Optical density analysis of A1AT. The columns represent mean values, error bars show standard deviation (SD) of three independent experiments (*n* = 3). Real-time polymerase chain reactions and ELISA measurements were carried out in triplicate in each independent experiment. Statistical significance was determined by two-way ANOVA (considering the number of the categorical variables) followed by Scheffe’s post hoc test. Asterisk indicates *p* < 0.05 compared to the control. Cross marks *p* < 0.05 compared to the fractalkine treatment. Double cross shows *p* < 0.05 compared to the LPS or LTA treatments, respectively. Abbreviations: F5—fractalkine 5 ng/mL; F10—fractalkine 10 ng/mL; LPS EC—*E. coli* lipopolysaccharide; LPS PA—*P. aeruginosa* lipopolysaccharide; LTA SA—*S. aureus* lipoteichoic acid.

Hepcidin is the major iron regulatory peptide, which is controlled by various stimuli such as inflammation [29]. HAMP (preprohepcidin) mRNA expression increased in the case of fractalkine treatments in a concentration-dependent manner (Figure 4A). The addition of fractalkine together with LPS EC increased the HAMP mRNA level (F10 5.305 ± 0.49, *p* = 0.00013) compared to LPS EC treatment (2.825 ± 0.43). In the case of LPS PA (F5 4.085 ± 0.28, *p* = 0.0001; F10 6.42 ± 0.21) or LTA SA (F5 3.31 ± 0.28, *p* = 0.00011; F10 6.02 ± 0.28, *p* = 0.0001) and fractalkine co-treatments, fractalkine elevated HAMP levels in a concentration-dependent manner (Figure 4A) compared to LPS PA (1.215 ± 0.04) and LTA SA (2.41 ± 0.14) treatments. Interestingly, significant alterations were not found between fractalkine alone, LPS and LTA alone or co-treatments in hepcidin secretion, although all treatments showed significant hepcidin elevation compared to the control (Figure 4B).

Due to the above-mentioned contestable results, the A1AT regulating preprohepcidin-hepcidin maturation process was also analysed in the differently treated THP-1 cells. Fractalkine treatments significantly increased the A1AT protein level (F5 160.46 ± 8.21%, *p* = 0.0012; F10 133.49 ± 7.12%, *p* = 0.0035) compared to the control (101.42 ± 5.56%) (Figure 4C,D). The addition of fractalkine and LPS EC or LPS PA or LTA SA to the THP-1 cells did not affect the A1AT level compared to LPS or LTA treatments suggesting that in the case of co-treatments the effect of bacterial cell wall components prevailed (Figure 4C,D).

### 2.4. Fractalkine Modifies the Expression of Iron Transporter Proteins of the LPS and LTA Treated THP-1 Cells

**Figure 5:**
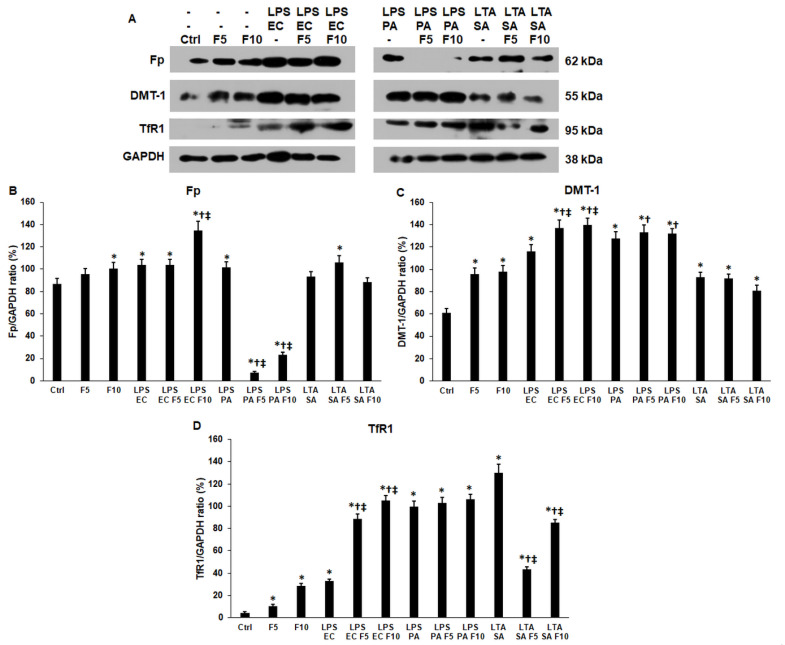
Western blot analyses of iron exporter ferroportin and iron importers divalent metal transporter-1 (DMT-1) and transferrin receptor 1 (TfR1) of *E. coli* or *P. aeruginosa* LPS and *S. aureus* LTA and/or fractalkine-treated THP-1 cells. The blots were probed with anti-Fp, anti-DMT-1 and anti-TfR1 polyclonal rabbit antibodies according to the manufacturer’s protocols. GAPDH was used as loading control on each membrane. (**A**) Western blot analyses of the examined proteins in THP-1 cells. (**B**–**D**) Optical density analyses of Fp, DMT-1 and TfR1 proteins. The columns represent mean values and error bars show standard deviation (SD) of three independent experiments (*n* = 3). Statistical significance was determined by two-way ANOVA (considering the number of the categorical variables) followed by Scheffe’s post hoc test. Asterisks indicate *p* < 0.05 compared to the control. Cross shows *p* < 0.05 compared to the fractalkine treatment. Double cross marks *p* < 0.05 compared to the LPS or LTA treatments, respectively. Abbreviations: F5—fractalkine 5 ng/mL; F10—fractalkine 10 ng/mL; LPS EC—*E. coli* lipopolysaccharide; LPS PA—*P. aeruginosa* lipopolysaccharide; LTA SA—*S. aureus* lipoteichoic acid.

Next, the iron transport proteins ferroportin (Fp), divalent metal transporter 1 (DMT-1) and transferrin receptor 1 (TfR1) were examined by Western blot. The protein level of the iron exporter Fp was significantly elevated using 10 ng/mL fractalkine (100.66 ± 4.1%, *p* = 0.018) compared to the control (86.95 ± 4.58%) (Figure 5A,B). Similarly, co-treatment of the THP-1 cells with LPS EC and 10 ng/mL fractalkine (134.88 ± 8.14%, *p* = 0.0049) significantly elevated the Fp level compared to LPS EC treatment (103.63 ± 5.12%) (Figure 5A,B). On the contrary, the addition of fractalkine and LPS PA (F5 7.35 ± 1.21%, *p* = 0.0001; F10 22.4 ± 1.89%, *p* = 0.00011) to the cells significantly downregulated the Fp level compared to LPS PA-treated (101.38 ± 5%) cells (Figure 5A,B). In the case of LTA SA and fractalkine co-treated cells, significant alteration of the Fp protein level was not found (Figure 5A,B).

The DMT-1 iron importer protein level significantly increased after fractalkine treatment (F5 95.74 ± 5.24%, *p* = 0.0007; F10 97.8 ± 5.49%, *p* = 0.0008) compared to the control (60.86 ± 4.23%). LPS EC and fractalkine co-treatment also increased the DMT-1 level (F10 139.53 ± 6.11%, *p* = 0.0079) compared to LPS EC (116.36 ± 5.59) suggesting that fractalkine further triggered the synthesis of the iron uptake protein (Figure 5A,C). LPS PA or LTA SA and fractalkine co-treatments did not show any significant effect on the DMT-1 levels compared to LPS PA or LTA-treated cells (Figure 5A,C).

The TfR1 iron importer protein level significantly increased after fractalkine treatment (F5 10.63 ± 1.21%, *p* = 0.0029; F10 28.57 ± 2%, *p* = 0.0001) compared to the control (4.34 ± 1%), similar to the DMT-1 level (Figure 5A,D). The same results were found in the case of LPS EC and fractalkine co-treatments (Figure 5A,D).

LPS PA and fractalkine co-treatments did not change the TfR1 levels significantly compared to LPS PA-treated cells. Interestingly, LTA SA and fractalkine co-treatments significantly reduced the TfR1 protein level (F5 43.66 ± 2.1%, *p* = 0.0001; F10 85.41 ± 2.89%, *p* = 0.0009) compared to LTA SA-treated (129.87 ± 7.89%) THP-1 cells (Figure 5A,D). Based on these findings, it is supposed that fractalkine acts in different ways on the iron transporters and therefore on the iron uptake and release of the cells.

### 2.5. Fractalkine Affects Differently the Expression of the Cytosolic and Mitochondrial Iron Storage Proteins of the LPS and LTA Treated THP-1 Cells

**Figure 6:**
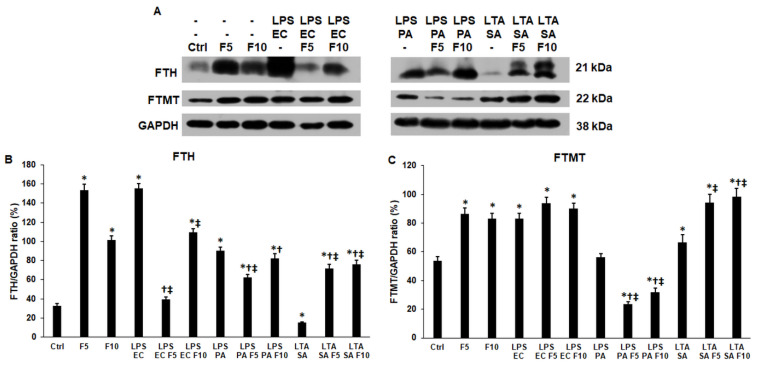
Western blot analyses of iron storage proteins ferritin heavy chain (FTH) and mitochondrial ferritin (FTMT) of *E. coli* or *P. aeruginosa* LPS and *S. aureus* LTA and/or fractalkine-treated THP-1 cells. The membranes were probed with anti-FTH and anti-FTMT polyclonal rabbit antibodies according to the manufacturer’s protocols. GAPDH was used as loading control of the WBs. (**A**) Western blot analyses of the examined proteins in THP-1 cells. (**B**,**C**) Optical density analyses of FTH and FTMT iron storage proteins. The columns represent mean values and error bars show standard deviation (SD) of three independent experiments (*n* = 3). Statistical significance was determined by two-way ANOVA (considering the number of the categorical variables) followed by Scheffe’s post hoc test. Asterisk marks *p* < 0.05 compared to the control. Cross indicates *p* < 0.05 compared to the fractalkine treatment. Double cross shows *p* < 0.05 compared to the LPS or LTA treatments, respectively. Abbreviations: F5—fractalkine 5 ng/mL; F10—fractalkine 10 ng/mL; LPS EC—*E. coli* lipopolysaccharide; LPS PA—*P. aeruginosa* lipopolysaccharide; LTA SA—*S. aureus* lipoteichoic acid.

The cytosolic and mitochondrial iron storage proteins were also examined to reveal the possible changes in the intracellular iron distribution. Both FTH and FTMT protein levels were significantly elevated after 24 h-long fractalkine treatments (FTH 153.64 ± 6.21%, *p* = 0.0001; FTMT 86.53 ± 4.21%, *p* = 0.0004) suggesting that fractalkine contributed to the regulation of iron storage (Figure 6A–C). When fractalkine was added together with LPS EC, the FTH level was reduced (39.58 ± 2.1%, *p* = 0.0001) compared to LPS EC (155.17 ± 5.47%) treatment and it was found that 5 ng/mL fractalkine was more effective on the FTH level (Figure 6A,B). In spite of this, FTMT levels did not change (Figure 6A,B). In the case of LPS PA and fractalkine co-treatments, only 5 ng/mL fractalkine significantly decreased the FTH protein level (62.5 ± 3%, *p* = 0.0005), but the FTMT protein level decreased (F5 23.43 ± 1.87%, *p* = 0.0001; F10 31.74 ± 3%, *p* = 0.0004) using both concentrations of fractalkine compared to LPS PA-treated cells (FTH 90.22 ± 3.58; FTMT 56.39 ± 2.54) (Figure 6A–C). Interestingly, in the case of fractalkine and LTA SA co-treatments, both FTH (71.56 ± 4.58%, *p* = 0.0001; 76.36 ± 4.23%, *p* = 0.0001) and FTMT (97.16 ± 5.88%, *p* = 0.0023; 98.36 ± 5.82%, *p* = 0.0021) protein levels significantly increased compared to LTA SA (FTH 15.15 ± 1%; FTMT 66.66 ± 5.21%) (Figure 6A–C). These results suggest that fractalkine not only affects the expression of iron storage proteins but acts in different ways on Gram-negative and Gram-positive bacterial cell wall component-treated THP-1 cells.

### 2.6. Fractalkine Alters the Levels of the Enzymes Responsible for the Heme and Iron Sulfur Cluster Syntheses of LPS and LTA Treated THP-1 Cells

**Figure 7:**
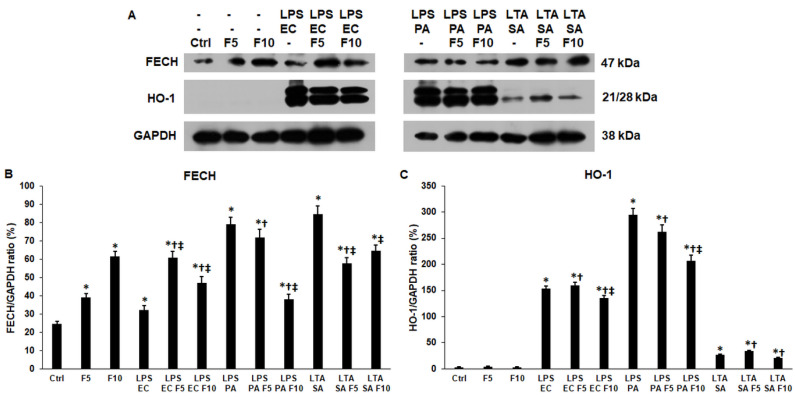
Western blot analyses of ferrochelatase (FECH) and heme oxygenase-1 (HO-1) of *E. coli* or *P. aeruginosa* LPS and *S. aureus* LTA and/or fractalkine-treated THP-1 cells. The membranes were probed with anti-FECH and anti-HO-1 polyclonal rabbit antibodies according to the manufacturer’s instructions. GAPDH was used as loading control of the WBs. (**A**) Western blot analyses of the examined proteins in THP-1 cells. (**B**,**C**) Optical density analyses of FECH and HO-1 proteins expressed by THP-1 cells. The columns represent mean values and error bars show standard deviation (SD) of three independent experiments (*n* = 3). Statistical significance was determined by two-way ANOVA (considering the number of the categorical variables) followed by Scheffe’s post hoc test. Asterisk marks *p* < 0.05 compared to the control. Cross shows *p* < 0.05 compared to the fractalkine treatment. Double cross indicates *p* < 0.05 compared to the LPS or LTA treatments, respectively. Abbreviations: F5—fractalkine 5 ng/mL; F10—fractalkine 10 ng/mL; LPS EC—E*. coli* lipopolysaccharide; LPS PA—*P. aeruginosa* lipopolysaccharide; LTA SA—*S. aureus* lipoteichoic acid.

Fractalkine treatments significantly increased the ferrochelatase (FECH) protein level (39.06 ± 2.11%, *p* = 0.0005; 61.52 ± 2.54%, *p* = 0.0001) compared to the control (24.61 ± 1.23%), while the heme oxygenase-1 (HO-1) level did not change (Figure 7A–C). These proteins are involved in heme synthesis and degradation, respectively. In the case of LPS EC and fractalkine co-treatments, the FECH protein level was significantly elevated (F5 60.86 ± 3.3%, *p* = 0.0003; F10 47.11 ± 3.11%, *p* = 0.0036) compared to LPS EC (32.2 ± 2.49%), and the HO-1 level reduced but only using 10 ng/mL fractalkine (135.25 ± 4.51%, *p* = 0.0158) (Figure 7A–C). In the case of LPS PA and fractalkine co-treatments, only 10 ng/mL fractalkine caused a significant decrease in both FECH (37.93 ± 2.71%, *p* = 0.0001) and HO-1 (206.89 ± 11.2%, *p* = 0.0008) protein levels (Figure 7A–C). By adding LTA SA and fractalkine together to THP-1 cells, we found a reduced FECH level (F5 57.69 ± 3.1%, *p* = 0.0011; F10 64.47 ± 3.2%, *p* = 0.0033) and invariable HO-1 level compared to LTA SA-treated (FECH 84.44 ± 4.62%) cells (Figure 7A–C). The findings show that fractalkine increases heme synthesis in the case of LPS EC but decreases it in the case of LPS PA and LTA SA treatments.

### 2.7. Fractalkine Changes the Heme Concentration and Iron Content of the Activated THP-1 Cells

**Figure 8:**
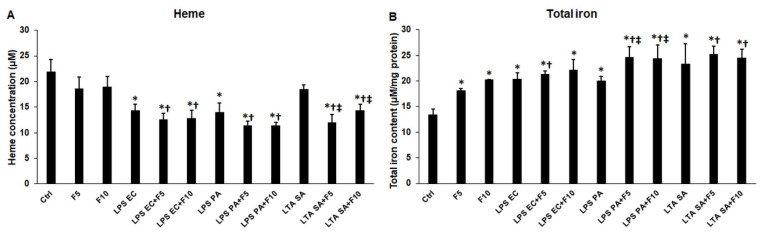
Determinations of heme concentration (**A**) and total iron content (**B**) of *E. coli* or *P. aeruginosa* LPS and *S. aureus* LTA and/or fractalkine-treated THP-1 cells. Heme concentration of the THP-1 cells was measured using a Heme Assay Kit. Iron content of the cells was determined using a colorimetric ferrozine-based assay and it was expressed as µM iron/mg protein. The columns represent mean values and error bars indicate standard deviation (SD) of three independent experiments (*n* = 3). The heme and total iron measurements were carried out in quadruplicate in each independent experiment. Statistical significance was determined by two-way ANOVA (considering the number of the categorical variables) followed by Scheffe’s post hoc test. Asterisk marks *p* < 0.05 compared to the control. Cross shows *p* < 0.05 compared to the fractalkine treatment. Double cross indicates *p* < 0.05 compared to the LPS or LTA treatments, respectively. Abbreviations: F5—fractalkine 5 ng/mL; F10—fractalkine 10 ng/mL; LPS EC—*E. coli* lipopolysaccharide; LPS PA—*P. aeruginosa* lipopolysaccharide; LTA SA—*S. aureus* lipoteichoic acid.

To reveal if alterations of the levels of the examined proteins implicated in iron utilization of the THP-1 cells, the heme concentration and total iron content of the THP-1 cells were measured after each treatment. Fractalkine treatment alone did not significantly affect the heme concentrations of the cells, but increased the total iron content (F5 18.15 ± 0.38 μM/mg, *p* = 0.0001; F10 20.31 ± 0.04 μM/mg, *p* = 0.0001) compared to control cells (13.44 ± 1.04 μM/mg) (Figure 8A,B). Neither LPS EC (12.59 ± 1.2 μM, *p* = 0.0543; F10 12.86 ± 1.48 μM, *p* = 0.0546) nor LPS PA (F5 11.44 ± 0.82 μM, *p* = 0.052; F10 11.48 ± 0.56 μM, *p* = 0.054) and fractalkine co-treatments altered the heme concentrations compared to LPS treatments alone (LPS EC 14.35 ± 1.14 μM; LPS PA 14.01 ± 1.81 μM) (Figure 8A), but a significant reduction in heme concentration was found when LTA SA and fractalkine (F5 12.05 ± 1.54 μM, *p* = 0.0001; F10 14.39 ± 1.12 μM, *p* = 0.0001) were added together to the THP-1 cells compared to the LTA SA treatment (18.48 ± 0.86 μM) (Figure 8A). A significant elevation of total intracellular iron content was found only in the case of LPS PA and fractalkine co-treatments (F5 24.63 ± 2.1 μM/mg, *p* = 0.0022; F10 24.46 ± 2.58 μM/mg, *p* = 0.0027), although a slight increment also appeared in the case of LTA SA and fractalkine co-treatments (F5 25.32 ± 1.51 μM/mg, *p* = 0.1688; F10 24.57 ± 1.58 μM/mg, *p* = 0.2314) (Figure 8B).

## 3. Discussion

Fractalkine is the only member of the CX3C subclass of chemokines [10]. Fractalkine is a unique transmembrane molecule expressed by many cells, such as neurons, glial cells immune cells and epithelial cells of the lung, kidneys and intestines and endothelial cells of the blood vessels [9]. This type of fractalkine, the membrane-bound form, acts as an adhesion molecule for macrophages and leukocytes [11,14,16]. The membrane-bound fractalkine can be cleaved by ADAM10 or ADAM17 to produce the soluble form of fractalkine [30,31]. It has been proven that fractalkine production increases at inflammatory conditions in several cell types [32,33,34].

Fractalkine binds to and activates its receptor, CX3CR1, triggering the action of distinct intracellular signalling pathways involving MAPK, PLC and NFκB. CX3CR1 is expressed by different inflammatory cell types such as microglia, lymphocytes and monocytes/macrophages [8,20,21]. The activation of the fractalkine–CX3CR1 axis regulates pro-inflammatory cytokine synthesis, cell growth, differentiation and proliferation [22,23].

Monocytes/macrophages can be activated by bacterial compounds such as bacterial cell wall components obtained from both Gram-negative (LPS) and Gram-positive (LTA) bacterial strains [2,35]. It has been proven that LPS or LTA inflammatory mediators from various sources affect differently both the inflammatory response and iron metabolism [15,36].

In the present work, we examined the effect of soluble fractalkine on the pro-inflammatory cytokine production and on the iron metabolism of human monocyte/macrophage THP-1 cells activated by *E. coli* LPS, *P. aeruginosa* LPS and *S. aureus* LTA.

Fractalkine alone did not affect cytokine secretion of the cells only in the presence of the bacterial cell wall components, suggesting that soluble fractalkine does not work as a direct activator of the monocytes.

In the case of co-treatments with fractalkine and the three different bacterial cell wall components, fractalkine modified differently the pro-inflammatory cytokine secretion: IL-1β, IL-6, TNFα and GM-CSF levels altered but IL-8 and IL-2 production showed less or no variation. The reason for this is perhaps that IL-8 acts as a chemoattractant of the immune cells, which may not be affected by fractalkine, while LPS triggers but LTA inhibits IL-2 production of T cells and they do not influence macrophage IL-2 expression [37,38]. Fractalkine increased GM-CSF secretion of the LPS as well as LTA-activated THP-1 cells in a concentration-dependent manner, suggesting that upon bacterial infection fractalkine may contribute to the proliferation and survival of macrophages [39].

IL-1β and IL-6 secretions showed an opposite alteration compared to TNFα production in the case of co-treatments: IL-1β and IL-6 levels decreased in each type of co-treatments; meanwhile, TNFα secretion increased in the case of the two different LPS and fractalkine co-treatments but was reduced in the case of LTA/fractalkine co-treatments. Comparing the reduction rate of IL-1β and IL-6 secretions, it can be seen that the presence of fractalkine was more effective on IL-6 than IL-1β. This can be explained by the fact that IL-1β undergoes a posttranslational maturation; therefore, the cells can still secrete IL-1β even though the mRNA expression has already decreased [40]. It can also be concluded that fractalkine may act differently on the regulation of TNFα expression; moreover, the type of inflammatory mediator (whether LPS or LTA) also influences the inflammatory response of THP-1 cells [36]. We also examined the relative mRNA expression of one additional M1 (INFγ) and four M2 (IL-4, IL-10, TGFβ and CD163) macrophage markers to reveal the possible effects of fractalkine on the polarization of THP-1 cells. Significant alterations were not found in the mRNA levels (Supplementary Figure S1).

The above observations can be the results of the alteration in the activity of the fractalkine/CX3CR1 axis and the downstream signalling cascades [22,23]. CX3CR1 directly regulates PLC, NFκB and MAPK pathways, but also controls the action of STAT3 and Nrf2/Keap-1 transcription factors via MAPK and NFκB [22,23]. Moreover, Nrf2 can modify the activity of the NFκB pathway [41]. The pro-inflammatory cytokine synthesis of THP-1 cells can be affected by these complex signalling interactions. On the other hand, pro-inflammatory cytokines such as IL-6, IL-1β or TNFα as well as NFκB and MAPK pathways and STAT3 regulate hepcidin synthesis, the main iron regulatory hormone [42,43,44]. Iron metabolism and inflammation are presumably interlinked via the fractalkine/CX3CR1 axis. However, fractalkine without an additional inflammatory signal did not change the activation of the downstream signalling pathways; it was able to modify the expression level of the signalling proteins at inflammation mediated by LPS or LTA. Moreover, it has been revealed that fractalkine has distinct effects on the signalling proteins depending on the inflammatory mediator used for the activation of THP-1 cells. The CX3CR1 protein level increased in the case of fractalkine and LPS EC co-treatments but was not modulated using LPS PA together with fractalkine. Moreover, THP-1 cells treated with LTA SA showed a decreased CX3CR1 level.

The NFκB p50 and p65 protein levels followed the alterations of the CX3CR1 level in the case of fractalkine and LPS EC and LPS PA co-treatments, but not in the LTA SA treatment. These distinct alterations may also be affected by TLR4 and TLR2, whose receptors are activated by LPS or LTA and play a role as regulators of the NFκB pathway [7]. We found similar discrepancies by the examination of PSTAT3 and the Nrf2/Keap-1 transcription activators: the PSTAT3 level after LPS EC/fractalkine co-treatment was consistent with NFκB protein levels; meanwhile, the additional treatments did not change PSTAT3 expression. The Nrf2/Keap-1 levels increased in the case of LPS PA or LTA SA and fractalkine co-treatments even though the CX3CR1 level did not change or decrease. These controversial results can be explained by the crosstalk between PSTAT3, MAPK, NFκB and Nrf2 signalling and by the possible convergence of CX3CR1 and TLR pathways [22,41,45].

Inflammation is one of the most important regulators of iron metabolism [29]. Synthesis of hepcidin, an iron regulatory hormone, is controlled by pro-inflammatory cytokines such as IL-6 and TNFα via the JAK/STAT3 and NFκB signalling pathways and can be also modified by MAPK signalling [42,43,44]. Hepcidin production is regulated by the fractalkine/CX3CR1 axis and via the aforementioned signalling cascades [8]. Hepcidin is transcribed as a preprohepcidin mRNA, which matures post-translationally into hepcidin by proteolytic cleavage [46]. The maturation process of hepcidin is regulated by the A1AT serine protease inhibitor [47]. Although the preprohepcidin mRNA levels increased with different rates of the distinct treatments, significant alterations in hepcidin secretion were not detected between fractalkine alone, LPS and LTA alone or co-treatments. Based on the A1AT protein levels, it can be supposed that the presence of the protease inhibitor in the cells inhibits hepcidin maturation, even though the cells synthesize more preprohepcidin mRNA molecules.

The increased hepcidin level may contribute to iron retention in the cells by decreasing iron export via inhibiting the ferroportin iron exporter protein even by activation of the internalization of Fp or by blocking the transport channel of the protein [48,49]. The presence of fractalkine and/or the inflammatory signal in the THP-1 cells may also assist in iron sequestration via DMT-1 or TfR1 iron importers. Unlike pro-inflammatory cytokine production, fractalkine alone was able to alter the expression of iron transporters and modified their levels in the case of co-treatments suggesting that fractalkine may have a crucial role in the elimination of iron from the circulation in the case of bacterial infections [36]. The total intracellular iron content of the differently treated cells was in correlation with the protein expression levels of the examined iron transporters Fp, DMT-1 and TfR1. A significant elevation of total intracellular iron content was found in the case of LPS PA and fractalkine co-treatments, and a slight increment also appeared in LTA SA/fractalkine co-treatments. These results are supported by the downregulation of Fp and normal expression levels of DMT-1 and TfR1 in the case of LPS PA/fractalkine co-treatments; normal expression of Fp and DMT-1 in the case of LTA SA/fractalkine co-treatments. The LPS EC/fractalkine co-treated cells did not show elevated intracellular iron content which may be due to the increased Fp level, which might antagonize the increased DMT-1 and TfR1 levels.

In parallel with the changes in the iron content of the cells, the expression of the cytosolic and mitochondrial iron storage proteins FTH and FTMT was also modulated. Fractalkine treatment alone increased both FTH and FTMT protein levels of THP-1 cells, suggesting that fractalkine contributed to the regulation of iron storage, as well. Furthermore, it has been revealed that fractalkine acts in different ways on Gram-negative and Gram-positive bacterial cell wall component-treated THP-1 cells: in the case of LPS PA/fractalkine co-treatments, both FTH and FTMT protein levels decreased; in the case of LTA SA/fractalkine co-treatments, both FTH and FTMT protein levels significantly increased. The decreased level of FTH in the case of the two types of LPS and fractalkine co-treatments may be the consequence of the elevated TNFα secretion acting as a regulator of FTH expression [50]. Moreover, FTH can trigger the expression of IL-6 and IL-1β production via phosphorylation of NFκB/p65 and MAPK [51,52]. Based on these data, it seems that fractalkine decreases FTH expression when it is utilized together with LPS, and as a consequence, reduces the pro-inflammatory cytokine IL-6 and IL-1β secretion.

Alterations in the iron distribution inside cells may vary mitochondrial iron utilization such as heme synthesis and degradation [53]. Ferrochelatase and heme oxygenase-1 levels were modified in the case of co-treatments showing that fractalkine increases heme synthesis in the case of LPS EC/fractalkine but decreases it in the case of LPS PA/fractalkine or LTA SA/fractalkine co-treatments. Interestingly, these observations can be supported by heme concentration measurements only in the case of LTA SA/fractalkine co-treatments with a decreased heme concentration. The invariable heme concentration of the LPS EC/fractalkine and LPS PA/fractalkine co-treated THP-1 cells may be the effect of the shift in FTMT protein levels and the mitochondrial iron availability. On the other hand, HO-1 expression is also regulated by the NFκB and Nrf2 and PSTAT3 transcription factors, which are under the control of CX3CR1 and TLRs [54,55,56,57]. Moreover, heme degradation is the classical function of HO-1; it also possesses enzyme-independent activity with which HO-1 is able to regulate transcription factors such as NFκB, STAT3 and Nrf2 [58].

**Figure 9:**
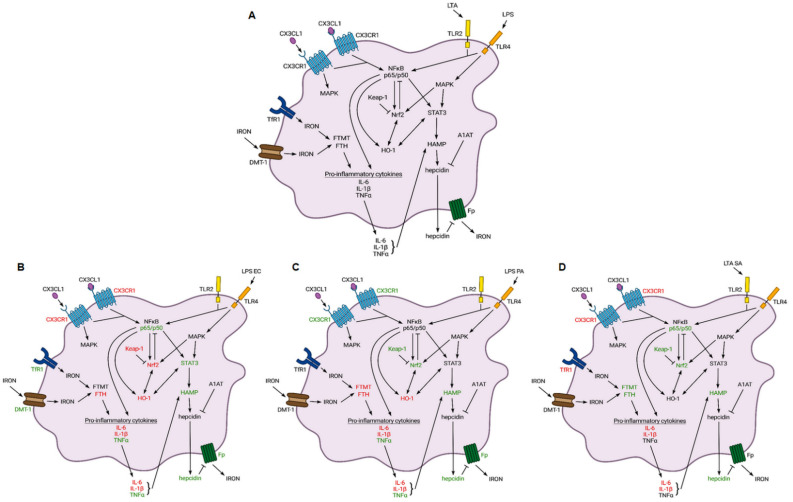
Cell signalling pathways regulated by CX3CR1 and TLR receptors and regulation of iron metabolism of THP-1 cells. (**A**) Fractalkine binds to CX3CR1 and activates NFκB and MAPK pathways. The same signalling pathways are regulated by TLRs. NFκB transcription factors regulate the transcription of Nrf2, STAT3, HO-1 and pro-inflammatory cytokines including IL-1β, IL-6 and TNFα. Meanwhile, Nrf2, which is inhibited by Keap-1, provides feedback on NFκB and HO-1 transcription. MAPK also modifies the activity of Nrf2 and STAT3 transcription factors by phosphorylation. PSTAT3 and pro-inflammatory cytokines increase HAMP transcription. Maturation of hepcidin is regulated by A1AT. TfR1 and DMT-1 function as iron importers and Fp act as iron exporter transmembrane protein. Fp is controlled by hepcidin. FTH and FTMT work as the cytosolic and mitochondrial iron storage proteins. (**B**) Alterations of the levels of signalling proteins and iron metabolism-related proteins in the presence of soluble fractalkine in *E. coli* LPS activated THP-1 cells. (**C**) Alterations of the levels of signalling proteins and iron metabolism-related proteins in the presence of soluble fractalkine in *P. aeruginosa* LPS activated THP-1 cells. (**D**) Alterations of the levels of signalling proteins and iron metabolism related proteins in the presence of soluble fractalkine in *S. aureus* LTA activated THP-1 cells. Green colour marks elevation, red colour indicates reduction in the expression levels of the examined proteins.

According to our results, soluble fractalkine acts both on the pro-inflammatory cytokine production and on the iron metabolism of THP-1 cells, activated by *E. coli* LPS, *P*. *aeruginosa* LPS and *S. aureus* LTA. It has been proven that the effect of fractalkine depends on the type of inflammatory mediator. Fractalkine acts via the CX3CR1 receptor regulating distinct intracellular signalling pathways NFκB, PLC and MAPK, and additional downstream signalling proteins such as STAT3 and Nrf2. The effect of fractalkine also modifies the action of LPS and LTA via the TLR-regulating NFκB and MAPK pathways (Figure 9).

Fractalkine attenuates the secretion of IL-6 and IL-β pro-inflammatory cytokines while triggering TNFα secretion in the presence of LPS but not LTA. According to these findings, it seems that fractalkine may influence the survival and apoptosis of macrophages via TNFα [59]. The pro-inflammatory cytokines, as well as fractalkine, are strong stimuli of hepcidin synthesis, which regulates iron homeostasis by inhibiting iron release and triggering iron retention of the cells. Changes in the protein levels of iron transporters and iron storage proteins mediated by fractalkine may contribute to the inhibition of bacterial proliferation by the elimination of iron from the environment [60]. In the present work, the THP-1 human monocyte/macrophage cell line was used in the experiments; therefore, further examinations of LPS/LTA-induced inflammatory animal models are needed for elucidating the systemic effect of soluble fractalkine on inflammation and on the regulation of iron homeostasis.

In summary, we propose that fractalkine operates as a link between inflammation and iron metabolism of macrophages. The impact of fractalkine on the cells depends on the type of bacterial infections (Gram-negative or Gram-positive) and also on the bacterial strains. Based on the results, we can gain a deeper insight into the role of soluble fractalkine in the regulation of the innate immune system.

## 4. Materials and Methods

### 4.1. Cell Culture and Treatments

The THP-1 human monocyte cell line, purchased from ATCC (TIB-202), was cultured in RPMI-1640 medium with l-Glutamine (Lonza Ltd., Basel, Switzerland) supplemented with 10% fetal bovine serum (FBS, EuroClone S.p.A, Pero, Italy) and 1% penicillin–streptomycin (P/S 10K/10K, Lonza Ltd., Basel, Switzerland). The activation of the cells was carried out using lipopolysaccharide obtained from *E. coli* and from *P. aeruginosa* Gram-negative bacterial strains (*E. coli 055:B5*, *P. aeruginosa 10*, Merck Life Science Kft., Budapest, Hungary) and lipoteichoic acid was purified from *S. aureus* Gram-positive bacterial strain (Merck Life Science Kft., Budapest, Hungary). LPS and LTA (1 mg/mL) and soluble fractalkine (100 µg/mL; Shenandoah Biotechnology Inc., Warminster, PA, USA) were solubilised in ultrapure distilled water (Merck Life Science Kft., Budapest, Hungary). The experiments were carried out at 37 °C in a humidified in vitro cell culture incubator containing 5% CO_2_. The THP-1 cells (5 × 10^5^) were plated onto 6-well uncoated cell culture plates (Greiner Bio-One Hungary Kft., Mosonmagyarovar, Hungary) and were rested for 24 h before the treatments. The cells were treated with 5 and 10 ng/mL of fractalkine alone for 24 h or 1 µg/mL LPS or LTA alone for 24 h. In the case of co-treatments, the same amount of fractalkine and LPS or LTA were added to the cells at the same time for 24 h. Untreated THP-1 cells were used as controls. The utilized concentrations of LPS/LTA and fractalkine were determined based on our previous article [36].

### 4.2. ELISArray

THP-1 cells were treated according to the aforementioned protocol. After 24 h, the cells were pelleted by centrifugation and the supernatants of the same treatments were pooled. The pooled samples were obtained from four independent experiments. Pro-inflammatory cytokine production of the THP-1 cells was monitored using the Human Pro-inflammatory Cytokine ELISArray (Qiagen Inc., Hilden, Germany) specific for IL-1β, IL-2, IL-6, IL-8, TNFα and granulocyte-monocyte colony-stimulatory factor (GM-CSF) according to the manufacturer’s instructions. Optical densities were measured at 450 nm using a MultiSkan GO spectrophotometer (Thermo Fisher Scientific Inc., Waltham, MA, USA).

### 4.3. Enzyme-Linked Immunosorbent Assay (ELISA)

After the treatments, the supernatants of differently treated and control THP-1 cells were collected and stored at −80 °C until the ELISA measurements. The IL-6, IL-1β and TNF-α pro-inflammatory cytokine concentrations of the culture media were determined with ELISA kits specific for human IL-1β, IL-6, and TNFα (Thermo Fisher Scientific Inc., Waltham, MA, USA). The secreted mature hepcidin content of the samples was measured with the Human Hepcidin Quantikine ELISA Kit (Bio-Techne R&D Systems Kft., Budapest, Hungary). All measurements were performed in triplicate according to the protocols of the manufacturers.

### 4.4. Real Time PCR

The cells were treated as previously described. After the incubation period, the cells were harvested and then total RNA was isolated from the cell pellets using the Aurum Total RNA Mini Kit (Bio-Rad Inc., Hercules, CA, USA). The RNA content of the samples was determined using a MultiSkan GO spectrophotometer (Thermo Fisher Scientific Inc., Waltham, MA, USA). Then, 200 ng total RNA from each sample was used for cDNA synthesis using the iScript Select cDNA Synthesis Kit (Bio-Rad Inc., Hercules, CA, USA). The real-time PCR reaction was carried out in 20 µL of total volume in a CFX96 One Touch Real-Time PCR System (Bio-Rad) using the SYBR Green protocol (iTaq Universal SYBR Green Reagent Mix; Bio-Rad Inc., Hercules, CA, USA). For the determination of the relative mRNA expression level (fold change) of the gene of interest, the β-actin housekeeping gene was used for normalization of the reaction and we utilized the Livak (∆∆Ct) method for the calculation using Bio-Rad CFX Maestro 1.1. software (Bio-Rad Inc., Hercules, CA, USA). The mRNA expression levels of the untreated control cells were considered as 1. The nucleotide sequences of the primers utilized for PCR can be seen in Table 1.

### 4.5. Western Blot

The THP-1 cells were collected by centrifugation after the treatments. Then, the cells of each sample were lysed with 150 µL of ice-cold lysis buffer (50 mM Tris-HCl, pH 7.4, 150 mM NaCl, 0.5% Triton-X 100) containing a complete mini protease inhibitor cocktail (Roche Ltd., Basel, Switzerland). Protein contents were determined using the DC Protein Assay Kit (Bio-Rad Laboratories, Hercules, CA, USA) and an equal amount of protein from each sample was separated in 10% or 12% polyacrylamide gels. Electrophoresis was carried out in a Mini Protean Tetra Cell equipment (Bio-Rad Laboratories, Hercules, CA, USA). The gels were transferred by electroblotting to nitrocellulose membranes (Pall AG, Basel, Switzerland). All membranes were blocked with a blocking solution containing 5% (*w*/*v*) non-fat dry milk (Bio-Rad Laboratories., Hercules, CA, USA) for 1 h at room temperature with gentle shaking. The membranes were incubated for 1 h at room temperature with the following polyclonal rabbit antibodies: anti-fractalkine receptor (CX3CR1) IgG (1:1000; Merck Life Science Kft., Budapest, Hungary), anti-ferroportin (Fp) IgG (1:1000; Bio-Techne, Minneapolis, MN, USA), anti-divalent metal transporter 1 (DMT-1) IgG (1:1000; Thermo Fisher Scientific Inc., Waltham, MA, USA) anti-transferrin receptor 1 (TfR1) IgG (Thermo Fisher Scientific Inc., Waltham, MA, USA), anti-alpha-1 antitrypsin (A1AT) IgG (1:1000; Thermo Fisher Scientific Inc., Waltham, MA, USA), anti-mitochondrial ferritin (FTMT) IgG (1:1000, Thermo Fisher Scientific Inc., Waltham, MA, USA) and anti-ferrochelatase (FECH) IgG (1:1000; (Thermo Fisher Scientific Inc., Waltham, MA, USA). Overnight incubation at 4 °C was used in the case of the following polyclonal rabbit antibodies: anti-NFκB/p50 IgG (1:1000; Cell Signaling Technology Europe, Leiden, The Netherlands), anti-NFκB/p65 IgG (1:1000; Cell Signaling Technology Europe, Leiden, The Netherlands), anti-phospho-STAT3 IgG (1:2000; Cell Signaling Technology Europe, Leiden, The Netherlands), anti-Nrf2 IgG (1:2000; Cell Signaling Technology Europe, Leiden, The Netherlands), anti-Keap-1 IgG (1:1000; Cell Signaling Technology Europe, Leiden, The Netherlands), anti-ferritin heavy chain (FTH) IgG (1:1000; Cell Signaling Technology Europe, Leiden, The Netherlands) and anti-heme oxygenase 1 (HO-1) IgG (1:1000; Cell Signaling Technology Europe, Leiden, The Netherlands). Glycerin aldehyde phosphate dehydrogenase (GAPDH) (anti-GAPDH IgG, 1:3000; Merck Life Science Kft., Budapest, Hungary) was used as a loading control for the Western blots. For the secondary antibody, horseradish peroxidase (HRP)-linked goat anti-rabbit IgG was used (1:3000; Bio-Rad Laboratories, Hercules, CA, USA) for 1 h at room temperature. For the development of WBs, we used traditional colourimetric detection using Fuji medical X-ray film (Fujifilm Corporation, Tokyo, Japan) and WesternBright ECL chemiluminescent substrate (Advansta Inc., San Jose, CA, USA). The optical density of the protein bands was determined by ImageJ software [61] and expressed as a percentage of the target protein/GAPDH ratio.

### 4.6. Intracellular Total Iron and Heme Measurements

Intracellular total iron measurements were performed according to our previous protocol [14]. Briefly, THP-1 cells were lysed with 200 µL of 50 mM NaOH at room temperature for 2 h with gentle shaking. The samples were neutralized by adding 100 µL of 10 mM HCl. Then, the samples were mixed with the iron-releasing reagent (100 μL; 1.4 M HCl, 4.5% (*w*/*v*) KMnO_4_ in H_2_O) and incubated for 2 h at 60 °C. Finally, 30 µL of iron detection reagent (6.5 mM ferrozine; 6.5 mM neocuproine; 2.5 M ammonium acetate; 1 M ascorbic acid) was added to each tube for 30 min at room temperature. The absorbance was measured at 550 nm using a MultiSkan GO spectrophotometer (Thermo Fisher Scientific Inc., Waltham, MA, USA). The iron content was determined by utilizing an FeCl_3_ standard curve [62]. For normalization, the protein concentration of the samples was measured with the DC Protein Assay Kit (Bio-Rad Laboratories, Hercules, CA, USA). The iron content of each sample was expressed as µM iron/mg protein. The cellular heme concentration was measured using the Heme Assay Kit (Merck Life Science Kft., Budapest, Hungary) according to the manufacturer’s protocol. Briefly, the cells were lysed with 100 µL of ultrapure water at room temperature then 50 µL of each sample was mixed with 200 µL of Heme Reagent. After 5 min incubation at room temperature, the absorbance was measured at 400 nm using a MultiSkan GO spectrophotometer (Thermo Fisher Scientific Inc., Waltham, MA, USA). The heme concentration was calculated according to the manufacturer’s instructions and expressed as µM. Both the total iron content and heme concentration measurements were carried out in quadruplicate in three independent experiments.

### 4.7. Statistical Analysis

ELISArray was carried out by using samples from four independent experiments. ELISA measurements and real-time PCR determinations were carried out in triplicate in three independent experiments. The intracellular total iron measurements and heme concentration determinations were carried out in quadruplicate in three independent experiments. Western blots were representative of three independent experiments. In each figure legend, n corresponds to the number of independent experiments. Statistical analysis was performed using SPSS software (IBM Corporation, Armonk, NY, USA). Statistical significance was determined by two-way ANOVA (considering the number of the categorical variables) followed by Scheffe’s post hoc test. Data are shown as the mean ± standard deviation (SD). The results were considered statistically significant if the *p*-value was <0.05.

## Figures and Tables

**Table 1:** Primers used in the experiments.

Primer	Sequence 5′ → 3′
HAMP forward	CAGCTGGATGCCCATGTT
HAMP reverse	TGCAGCACATCCCACATC
β-actin forward	AGAAAATCTGGCACCACACC
β-actin reverse	GGGGTGTTGAAGGTGTCAAA

## Data Availability

Not applicable.

## Supplementary Materials

The following supporting information can be downloaded at: https://www.mdpi.com/article/10.3390/ijms23052629/s1.
