# Supplementary material for: Modulatory Effects of Fractalkine on Inflammatory Response and Iron Metabolism of Lipopolysaccharide and Lipoteichoic Acid-Activated THP-1 Macrophages
Source: Int J Mol Sci. 2022 Feb 27;23(5):2629. doi: 10.3390/ijms23052629 (PMC8910483; doi:10.3390/ijms23052629)
Supplement: Supplementary file 1 [file ijms-23-02629-s001.zip › ijms-1596158-supplementary/Supplementary material.pdf]

Modulatory Effects of Fractalkine on Inflammatory Response and Iron Metabolism of Lipopolysaccharide and Lipoteichoic Acid Activated THP-1 Macrophages

Edina Pandur*, Kitti Tamási, Ramóna Pap, Gergely Jánosa and Katalin Sipos

[1] Department of Pharmaceutical Biology, Faculty of Pharmacy, University of Pécs, Rókus Str. 2, H-7624 Pécs, Hungary, edina.pandur@aok.pte.hu; kitti.tamasi@aok.pte.hu; pap.ramona@pte.hu; janosa.gergely@gytk.pte.hu; katalin.sipos@aok.pte.hu
* Correspondence: edina.pandur@aok.pte.hu

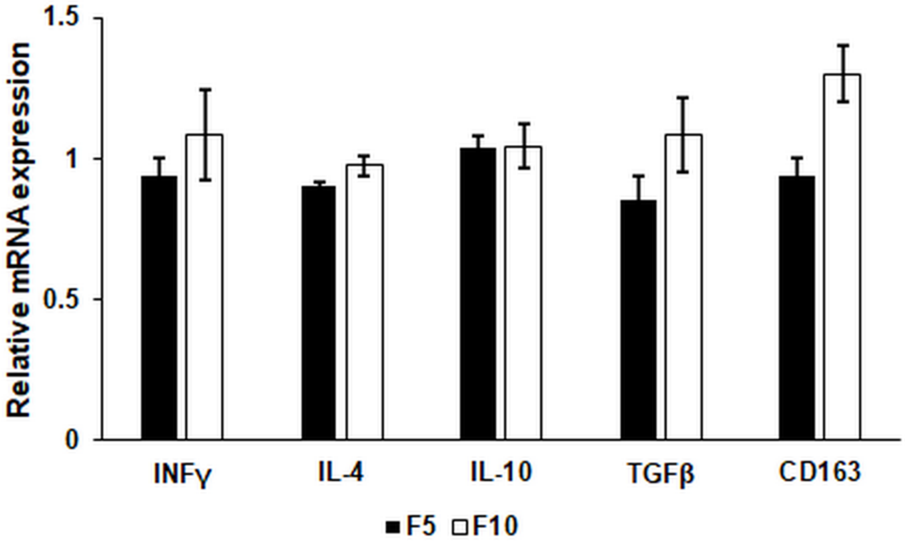

Supplementary Figure S1. Real time PCR measurements of INFγ M1 marker and IL-4, IL-10, TGFβ and CD163 M2 markers of fractalkine treated THP-1 cells. Real-time PCR was carried out with SYBR Green protocol and β-actin housekeeping gene was used as internal control for the normalization of the reaction. The relative expression of untreated control cells was regarded as 1. The columns represent mean values, error bars show standard deviation (SD) of three independent experiments (n = 3). Real time polymerase chain reactions were carried out in triplicate in each independent experiment. Statistical significance was determined by two-way ANOVA (considering the number of the categorical variables) followed by Scheffe's post hoc test. Significant changes were not found. Abbreviations: F5 - fractalkine 5 ng/mL; F10 - fractalkine 10 ng/mL.
